# ORFV infection enhances CXCL16 secretion and causes oncolysis of lung cancer cells through immunogenic apoptosis

**DOI:** 10.3389/fcimb.2022.910466

**Published:** 2022-07-25

**Authors:** Ruixue Wang, Jingying Mo, Xiaoshan Luo, Guixian Zhang, Fang Liu, Shuhong Luo

**Affiliations:** ^1^ Department of Basic Medical Sciences, School of Medicine, Foshan University, Foshan, China; ^2^ Department of Laboratory Medicine, School of Medicine, Foshan University, Foshan, China

**Keywords:** Orf virus (ORFV), lung cancer, apoptosis, immunogenic cell death (ICD), CXCL16

## Abstract

Oncolytic viruses have been emerging as a promising therapeutic option for cancer patients, including lung cancer. Orf virus (ORFV), a DNA parapoxvirus, can infect its natural ungulate hosts and transmit into humans. Moreover, the ORFV has advantages of low toxicity, high targeted, self-amplification and can induce potent Th1-like immunity. This study explored the therapeutic potential of ORFV infection for human lung cancer therapy and investigated the molecular mechanisms. We used a previously described ORFV NA1/11 strain and tested the oncolysis of ORFV NA1/11 in two lines of lung cancer cells *in vitro* and *in vivo*. Treatment of both cell lines with ORFV NA1/11 resulted in a decrease in cell viability by inducing cell cycle arrest in G2/M phase, suppressing cyclin B1 expression and increasing their apoptosis in a caspase-dependent manner. The ORFV NA1/11-infected lung cancer cells were highly immunogenic. Evidently, ORFV NA1/11 infection of lung cancer cells induced oncolysis of tumor cells to release danger-associated molecular patterns, and promoted dendritic cell maturation, and CD8 T cell infiltration in the tumors by enhancing CXCL16 secretion. These findings may help to understand the molecular mechanisms of ORFV oncolysis and aid in the development of novel therapies for lung cancer.

## Introduction

Oncolytic viruses (OVs) can replicate and cause cytotoxicity against human tumor cells, which release tumor antigens to induce long lasting anti-cancer memory T cell responses (Kaufman et al., 2015). Since the U.S. Food and Drug Administration has approved the first OV, Talimogene laherparepvec (T-VEC), a herpes simplex virus encoding GM-CSF, for treatment of melanoma, there are great interests in development of OV therapies for other types of cancers, such as head and neck cancer, pancreatic cancer, ovarian cancer, colorectal cancer, non-small cell lung cancer (Russell and Peng, 2018). Many research groups have tested a series of natural and engineered viruses for their potential as OVs both *in vitro* and *in vivo*, and several OVs are being tested in clinical trials (Kaufman et al., 2015).

Following infection, OVs can replicate and kill the recipient cells typically through multiple cell death pathways (Guo et al., 2014). Following infection with OVs, some recipient cells undergo programmed cell death and the dying cells can be recognized and phagocytosed by antigen presenting cells (APCs), such as dendritic cells (DCs) and macrophages, to induce antitumor T cell immunity. Simultaneously, the dying tumor cells undergo organellar and cellular stress and can secrete damage-associated molecular patterns (DAMPs), including calreticulin, adenosine triphosphate (ATP) and high-mobility group box 1 (HMGB1). Each of them acts as danger signals that are recognized by APCs, and drive antitumor T cell immunity, a process of immunogenic cell death (ICD) (Zhou et al., 2019). ICD is a potent antigenic source for DCs to cross-present antigen and active cytotoxic T lymphocytes.

Orf virus (ORFV) is a double stranded DNA parapoxvirus, and mainly infects sheep and goats, causing skin lesions, which can be transmitted to humans (Spyrou and Valiakos, 2015). ORFV has been developed as an antiviral vaccine (Baypamune^®^, Bayer; Zylexis^®^, Pfizer) in veterinary medicine. Interestingly, infection with living ORFV strain NZ2 from New Zealand inhibits the growth of melanoma and colorectal cancer by activating nature killer (NK) cells and stimulating secretion of IFN-γ and Granzyme B (Rintoul et al., 2012). Similarly, infection with inactivated ORFV strain D1701 from German can induce innate immune responses by activating NK and NKT cells, leading to potent antitumor activity in rodent models of cancers (Fiebig et al., 2011). Recently, infection with ORFV CF189, similar to ORFV NZ2 strain, can effectively kill different types of triple negative breast cancer (TNBC) cells in a time- and dose-dependent manner, which is associated with many NK cell infiltrates in the tumors (Choi et al., 2018). It is notable that activated DCs and macrophages can secrete many types of pro-inflammatory cytokines and chemokines, such as IFN-α and CXCL16 in the tumor environment. CXCL16 is an important chemokine and can be secreted by NK cells, DCs as well as various types of cancer cells **(** La Porta, 2012). CXCL16 is a chemotactic factor for CXCR6^+^ T cells, NK, NKT and plasma cells to enhance antitumor T cell immunity (Unutmaz et al., 2000 **;** Kim et al., 2001 **;** Kim et al., 2002 **;** Motsinger et al., 2002 **;** Nakayama et al., 2003 **;** La Porta, 2012).

The OVs are varying in infectivity, replication and oncolysis, which may trigger different immune responses. It is important to understand how an ORFV strain mediates ICD in a specific type of malignant tumor. Our previous study has isolated an ORFV NA1/11 strain (Li et al., 2012). In this study, we explored how ORFV NA1/11 infection of lung cancer cells triggered ICD *in vitro* and *in vivo*. Our data indicated that ORFV NA1/11 infection triggered the apoptosis of lung cancer cells and inhibited the growth of implanted lung tumors in mice by enhanced release of HMGB1, ATP and CXCL16 secretion to promote DCs activation and recruit CD8 T cells. Our findings may aid in design of new therapies for lung cancer.

## Materials and methods

### Cell culture

Ovine fetal turbinate (OFTU) cells were maintained in our laboratory and cultured in MEM medium containing 10% fetal bovine serum (FBS), 100 U/ml of penicillin and 100 μg/ml of streptomycin (complete medium). Human lung cancer A549 and mouse Lewis lung cancer (LLC) cells were obtained from the American Type Culture Collection (ATCC, Manassas, VA, USA), and cultured in 10% FBS PRIM-1640 (complete medium). All cells were cultured at 37°C in a humidified atmosphere of 5% CO_2_. Cell culture media, FBS, phosphate buffered saline (PBS) and antibiotics were from Gibco (Waltham, MA, USA).

### Propagation and titration of ORFV

To prepare virus stocks, OFTU cells (1 ×10^7^ cells/plate) were cultured in 10-cm plates overnight and after being washed with PBS, the cells were infected with ORFV NA1/11 at a multiplicity of infection (MOI) of 1 in Opti-MEM (Invitrogen, Carlsbad, CA, USA) for 2 hours. After the supernatants were removed, the cells were cultured in complete medium. Two days later, when most cells were infected, the cells and their supernatants were collected. The cells were subjected to three cycles of freeze/thaw to release virions. After being centrifuged at 1000 g for 10 minutes at 4°C, their supernatants were collected and centrifuged (16000 g at 4°C) for 60 minutes. The precipitates were re-suspended in PBS and stored at −80°C. The contents of viruses were titered in OFTU cells, as previously described (Li et al., 2012). Briefly, virus titration was obtained in OFTu cells by microscopic assessment of virus-specific cytopathogenic effects over a period of up to 8 days, and a 50% tissue culture infectious dose (TCID50) was determined according to the Spearman-Kaerber method.

### Cell viability assay

A549 and LLC cells (2×10^3^/well) were cultured in 96-well plates overnight and infected in triplicate with, or without, ORFV NA1/11 at MOIs (0.01, 0.05, 0.1, 0.5, 1, 5, 10) for indicated time points. During the last 1-4 hours of incubation, each well of cell was exposed to WST-8 solutions (Dojindo, Japan). The absorbance was measured at 450 nm.

### Cell cycle analysis

A549 and LLC cells were cultured in 6-well plates overnight and infected with, or without, ORFV NA1/11 at a MOI of 5 for 24 hours. The cells were analyzed for their cell cycle status using cell cycle staining kit (Multi Sciences, Hangzhou, China), according to the manufacturer’s instructions. Briefly, the harvested cells were fixed with 70% ethanol, permeabilized, treated with RNase A and stained with propidium iodide (PI) in the dark. The cells were analyzed by flow cytometry (CytoFLEX S, Beckman, USA). The percentages of cells in G0/G1, S, and G2/M phases were quantified using Flowjo software.

### Western blot assay

For the total protein, the different groups of cells were lysed in lysis buffer (KeyGEN biotech, Nanjing, China) and centrifuged. For fractionation of cytoplasmic proteins, lysis buffer (10 mM HEPES pH 7.9, 10 mM KCl, 0.1 mM EDTA, 1.5 mM MgCl2, 0.2% v/vNP40) was added to the cell pellet with occasional gentle pipetting and then the samples were centrifuged for the supernatant collected. After determining protein concentrations, the cell lysates (30 µg/lane) were separated by sodium dodecyl sulfate–polyacrylamide gel electrophoresis (SDS-PAGE) and transferred onto polyvinylidene difluoride (PVDF) membrane. After being blocked, the membranes were incubated with primary antibodies against Cyclin B1 (Cat# 4138, Cell Signaling Technology, Danvers, MA, USA), GAPDH (Cat# 2118S, Cell Signaling Technology), HMGB1(Cat# 66525-1-Ig, Proteintech, Rosemont, USA) and successively reacted with horseradish peroxidase (HRP)-conjugated secondary antibodies, followed by visualized using enhanced chemiluminescence, as previously described (Wang et al., 2017). The blots were analyzed using the Quantity one software.

### Cell apoptosis analysis

The impact of ORFV NA1/11 infection-induced cell apoptosis was quantified by flow cytometry using the Apoptosis Detection Kit (BD Biosciences, Mississauga, Ontario, Canada). In brief, the cells (4×10^5^ cells/well) were cultured in 6-well plates overnight and infected with, or without, ORFV NA1/11 at a MOI of 5 for 2 hours. After being washed, the cells were cultured in complete medium for 48 hours. The cells were stained with FITC-anti-Annexin-V and PI solution for 15 minutes in the dark. After being washed, the cells were analyzed by flow cytometry, and the data were analyzed using CytoExpert software (Beckman).

### Caspase activity measurements

Cells (1 × 10^4^ cells/well) were cultured in 96-well plates overnight and infected in triplicate with, or without, viruses at a MOI of 5 for 24 hours. The levels of caspase 3/7/8 activities in individual wells were measured by luminescent assay using the Caspase-Glo 3/7 kit (#G8091, Promega, Madison, WI, USA), caspase 8 assay kit (C1151, Beyotime, Shanghai, China) in a Synergy HTX Multi-Mode reader (BioTek, VT, USA).

### ATP measurements

ATP concentration was examined using an ATP assay kit (S0027, Beyotime, Shanghai, China), according to the manufacturer’s protocol. Briefly, the culture medium from each well were collected for the ATP assay. Culture medium were mixed with 100 μL of ATP detection solution and the levels of ATP in individual wells were detected by chemiluminescence using a Synergy HTX Multi-Mode reader.

### Isolation and culture of mouse splenocytes, DCs and T cells

Peripheral blood DCs were isolated from mice using the DC Isolation Kit (#DC2012MK, TBD Science, China), according to the manufacturer’s protocol. Briefly, mouse anticoagulated blood samples were reacted with Separate 1 and Separate 2 to make a gradient interface. After centrifugation 2000 g for 20 min, the DC-enriched second layer was carefully collected and the cells were washed, centrifuged and resuspended in complete medium. The isolated cells were cultured in complete medium. The purity of isolated DCs was examined by flow cytometry after being stained with fluorescent-anti-CD11c antibody (Cat# 561022, BD Biosciences).

CXCR6^-/-^ C57BL/6 mice were generated and kindly provided by Prof. Fang Liu (Department of Basic Medical Sciences, Foshan University). Splenic mononuclear cells were isolated from CXCR6^-/-^ and wide-type (WT) C57BL/6 mice and cultured in complete medium. The splenic T cells were isolated from mice by negative selection using EasySep Mouse T Cell Isolation Kit (#19851, Stemcell, VAN, Canada) following the manufacturer’s protocol. In brief, splenic mononuclear cells were passed through a 70-µm cell strainer to obtain single cell suspension. The cells (1.0 X 10^8^ cells/mL) were stained with 50 μL of cocktail reagents at room temperature for 10 min, and reacted with 75 μL of EasySep Rapidspheres in 2 mL RPIM-1640 for 2.5 min. The magnetic antibody-bound cells were loaded on the EasySep column. The unbound T cells were collected from the flow through. The purity of isolated T cells were stained with fluorescent-anti-CD3 (Cat# 562286, BD Biosciences) and analyzed by flow cytometry.

### *In vitro* co-culture of LLC cells and DCs

LLC cells (4×10^5^ cells/well) were infected with, or without, ORFV NA1/11 at a MOI of 5 for 2 hours in the bottom chamber of 12-well transwell plates (Corning, New York, USA) and washed with PBS. DCs (1×10^6^ cells/well) were added to the upper chamber of transwell plates and cultured for 24 hours. The mean fluorescence intensity (MFI) of MHC I (Cat# 562823, BD Biosciences), CD80 (Cat# 560523, BD Biosciences), CD86 (Cat# 560581, BD Biosciences) expression in the migrated DCs were analyzed by flow cytometry.

### *In vitro* co-culture of LLC cells, DCs and T cells

LLC cells (4×10^5^ cells/well) in the bottom chamber of 12-well transwell plates were infected with, or without, ORFV NA1/11 at a MOI of 5 for 2 hours and washed. DCs (1×10^6^ cells/well) and T cells (2×10^6^ cells/well) from WT and CXCR6^-/-^ mice were added in the upper chambers and co-cultured for 24 hours. The percentages of migrated T cells were analyzed by flow cytometry after staining the cells with fluorescent-anti-CD4 (Cat# 561830, BD Biosciences), or fluorescent-anti-CD8 (Cat# 557959, BD Biosciences) antibodies. In some experiments, the LLC cells were treated with, or without, 0.5 µg/ml of anti-CXCL16 for 2 hours before DCs and T cells were added into the upper chambers.

### Quantitative real-time PCR

ORFV024 gene of ORFV was cloned into the pET21b (+) plasmid to construct the recombinant plasmid pET21b-ORFV024. The pET21b-ORFV024 recombinant plasmid DNA was isolated and used as a template for qPCR detection of ORFV using the SYBR master mixture (#A25742, Thermo Scientific) in the Real-Time PCR platform (StepOne, ABI, USA), and a standard curve was established. Total DNA was extracted from the different groups of cells and analyzed by qPCR. The sequences of primers for ORFV024 amplification were as follows: Forward- AAGAATTCATGGCTTCCTACATC, Reverse- ATAAGCTTCTACACGTAAACCGTGTG.

### Phagocytosis assay

The LLC cells were labeled with 2.5 µM CFSE dye (Cat# 65-0850-84, Carlsbad, CA, USA) in PRIM-1640 medium for 15 min at 37°C and washed with complete medium. The LLC cells were infected with or without, ORFV NA1/11 (MOI=5). Subsequently, the CFSE-labeled tumor cells were co-cultured with peripheral blood DCs for 24 hours in a transwell plate. To determine the effect of phagocytosis by DCs, the DCs were stained with fluorescent-conjugated anti-CD11c monoclonal antibody. The percentages of DCs (CD11c^+^CFSE^+^) containing LLC cells in total DCs were analyzed by flow cytometry.

### ELISA

LLC cells were infected with, or without, ORFV NA1/11 at a MOI of 5 for 24 hours. The levels of CXCL16 in the supernatants of cultured cells were quantified by ELISA using a specific kit (Zikerbio, Shenzen, China) according to the manufacturer’s instruction. Briefly, the supernatant samples (50 μL each) were tested in triplicate and the captured CXCL16 was detected with HRP-labeled detection antibody. After being washed 5 times with washing buffer, each well was added with 50 μL of substrate A and B, followed by adding 50 μL of stopping solution. The optical density (OD) values were measured at 450 nm.

### Animal studies

All procedures involving animals and their care were conducted in accordance with the guidelines of the Institutional Animal Care and Use Committee of Foshan University (20210303-14). C57BL/6 WT mice or nude mice at 6 weeks of age were implanted subcutaneously with LLC cells (4 × 10^5^ cells/mouse) or A549 cells (3× 10^6^cells/mouse) in the right flank on day 0. When the tumors volumes reached 50-100 mm^3^, the mice were randomized and injected intratumorally with the vehicle PBS or ORFV NA1/11 strain (1× 10^7^ pfu) in 50 µl on days 5, 7, and 9 post implantations. The tumor volume (V) was measured longitudinally with a caliper and calculated using the following formula: V = A×B^2^×0.5 (A= long axis, B=short axis). At the end of the experiment, animals were euthanized when the tumor exceeded 20 mm in either dimension. The tumors were dissected and weighed. Some tumor and non-tumor tissues were fixed in 4% paraformaldehyde for subsequent histology and immunohistochemistry. Other parts of tumors were used for Western blot and flow cytometry.

### Histology and immunohistochemistry

The paraffin-embedded tumor and other tissue sections (4 µm) were routine-stained with hematoxylin and eosin. In addition, the sections were dewaxed, rehydrated and probed with ant-Ki67 (1:200 dilution, Cat# GB111141, Servicebio, Wuhan, China), cleaved caspase 3 (1:200 dilution, Cat# GB11532, Servicebio). The bound antibodies were detected with HRP-conjugated secondary antibodies and visualized using the immunohistochemical detection kit (#BD5001, Bioworld, Nanjing, China), followed by counterstaining with hematoxylin. The immunostaining signals were captured under a light microscope.

### Flow cytometry

The fresh tumor tissues were cut into small pieces, and digested with 2 mg/mL of collagenase D and 40 U/mL of DNase to obtain a single-cell suspension. To isolate lymphocyte, the single-cell suspension was subjected to lymphocyte isolation using the lymphocyte isolation solution (TBD, Tianjin, China). The isolated cells were stained with Viability Dye 506 (Cat# 62210-00, Biogems, CA, USA) and antibodies against CD45 (Cat# 553080, BD Biosciences, Mississauga, Ontario, Canada), CD3, CD4, CD8, MHC I, CD80, CD86, CD11c and granzyme B (REF# 12-8898-80, Invitrogen, Carlsbad, CA, USA). After being washed, the cells were analyzed by flow cytometry. The vital cells were gated and the frequency of each subset of cells was analyzed.

### Cytokine antibody array

Equal amount of tumor tissues from the different groups of mice were homogenized and after being centrifuged, the levels of cytokines in the tumor environment of each group of tumors were quantified by cytokine array using the semi−quantitative mouse cytokine antibody array kit (GSM-CAA-4000; RayBiotech), following the manufacturer’s protocol. In brief, the cytokine array slides were blocked with 100 μl of Sample Diluent and added in triplicate with 100 μl of standard cytokine (positive control) or individual samples. After being extensively washed, the bound cytokines were detected with biotinylated antibodies and reacted with Cy3 equivalent Dye−Streptavidin. The fluorescent signals were visualized through a laser scanner (InnoScan 300 Microarray Scanner; Innopsys). The fluorescent signals were analyzed by Q−Analyzer software (QAM-CAA-4000-SW).

### Statistical analysis

All numerical data are presented as mean ± standard deviation (SD). Statistical significance between two groups was analyzed by Student’s t test using GraphPad Prism 7.0. A one-way ANOVA was performed in multiple comparisons and a two-way ANOVA was used to detect the significant difference of tumor volume growth curves. A P-value of < 0.05 was considered statistically significant.

## Results

### Oncolytic activity of ORFV NA1/11 against lung cancer cells

**Figure 1:**
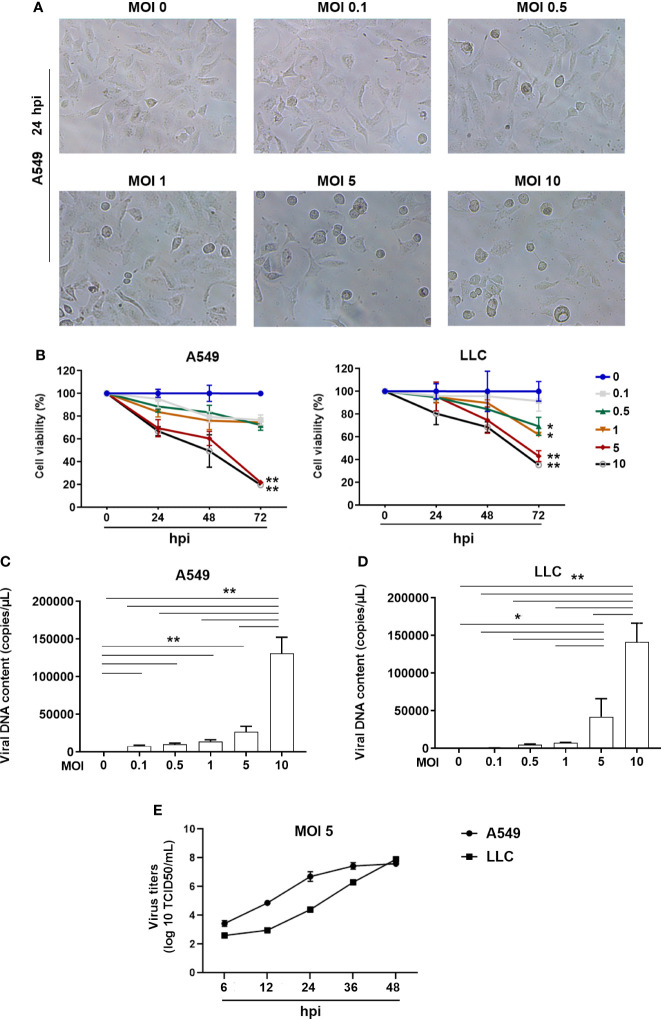
ORFV NA1/11 infection causes oncolysis of lung cancer cells. **(A)** Morphological changes in A549 cells following infection with, or without, ORFV NA1/11 at the indicated MOIs for 24 hours. **(B)** WST-8 assays measured the cell viability after infection in triplicate with, or without, ORFV NA1/11 at the indicated MOIs for the varying periods. The uninfected cells were designated as 100%. **(C, D)** qPCR evinced that the infected viruses replicated in lung cancer cells. Data are representative images or expressed as the mean ± SD of each group from three separate experiments. **(E)** Time course of viral replication in A549 and LLC cells after infection of ORFV (MOI 5). Data are representative images or expressed as the mean ± SD of each group from three separate experiments. *P<0.05, **P<0.01.

To investigate whether the ORFV NA1/11 could infect lung cancer cells, several lung cancer cell lines were infected with, or without, ORFV NA1/11 at a MOI of 5 for 48 hours. We found that infection with ORFV NA1/11 caused varying levels of cell death and A549, LLC and PC9 cells were more sensitive to ORFV NA1/11 infection than others (**Supplementary Figure 1A**). We chose A549 and LLC cells for subsequent studies. Following infection with ORFV NA1/11 at varying MOIs for 24 hours, we found that infection with ORFV NA1/11 at a MOI of 5 or higher caused obvious cytopathy in A549 cells, including cell becoming rounding, swelling and detachment (**Figure 1A**). Besides, with a recombinant virus ORFV NA1/11−GFP infection, the signal of GFP was visible under fluorescent microscope (**Supplementary Figure 1B**).

We measured cell viability of A549 and LLC cells after infection with different doses of ORFV NA1/11. We found that infection with different doses of ORFV NA1/11 decreased cell viability in a dose- and time-dependent manner in both A549 and LLC cells *in vitro* (**Figure 1B**). Furthermore, infection with ORFV NA1/11 at different doses promoted its replication in a dose-dependent manner in A549 and LLC cells (**Figures 1C, D**). Using titer determination, we found that ORFV did replicate in A549 and LLC cancer cells (**Figure 1E**). Collectively, these data indicated that ORFV NA1/11 effectively infected lung cancer cells and caused their death *in vitro*.

### ORFV NA1/11 infection arrests cell cycle in G2/M phase in lung cancer cells

**Figure 2:**
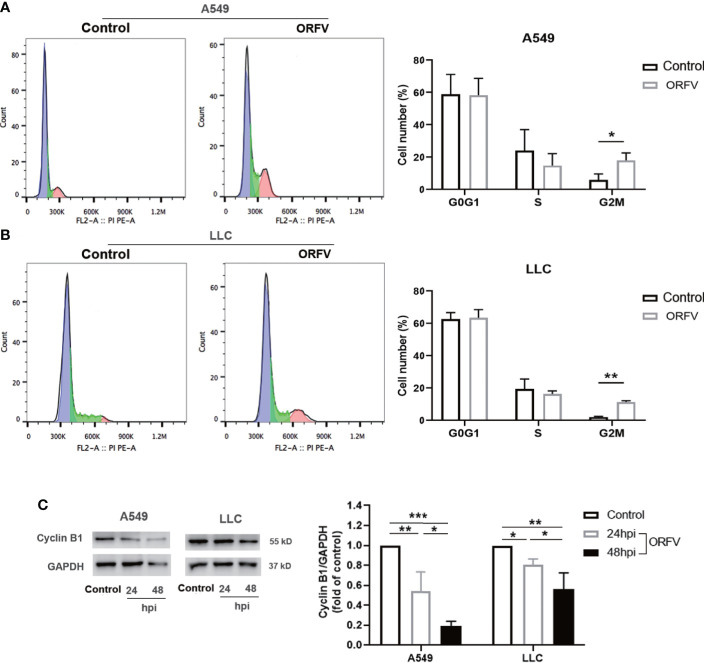
ORFV NA1/11 infection induces cell cycle arrest in A549 and LLC cells. **(A, B)** Flow cytometry analysis revealed that ORFV NA1/11 infection for 24 hours increased the frequency of cells in G2/M-phase, compared with uninfected cells (Control). **(C)** Western blot analysis of the relative levels of cyclin B1 expression in A549 and LLC cells following infection with, or without, ORFV NA1/11 (MOI 5) for indicated time points. Quantification was performed using Quantity one software. Data are representative images or expressed as the mean ± SD of each group from three separate experiments. *P<0.05, **P<0.01. ***P<0.001.

To gain a more detailed insight into the effect of ORFV NA1/11 infection on cell cycling, A549 and LLC cells were infected with, or without, ORFV NA1/11. One day later, their cell cycle status was analyzed by flow cytometry. As shown in **Figures 2A, B**, ORFV NA1/11 infection increased the percentages of lung cancer cells in the G2/M phase. Western blot analysis indicated that ORFV NA1/11 infection decreased the relative levels of cyclin B1 expression in both A549 and LLC cells relative to that in the controls (**Figure 2C**). Hence, ORFV NA1/11 infection induced cell cycle arrest in G2/M phase in lung cancer cells *in vitro*.

### ORFV NA1/11 infection induces the caspase-dependent apoptosis, but not necroptosis in lung cancer cells

**Figure 3:**
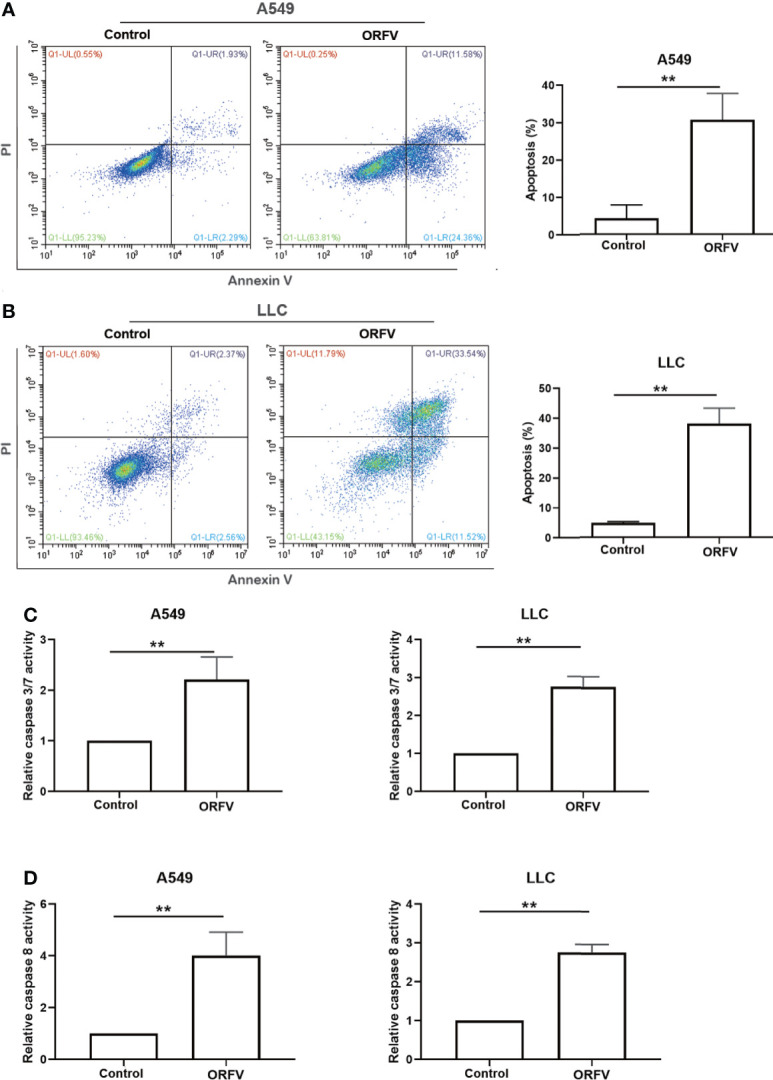
ORFV NA1/11 infection induces apoptosis of A549 and LLC cells. **(A, B)** Flow cytometry analysis of the percentage of apoptotic A549 and LLC cells following infection with, or without, ORFV NA1/11 for 48 hours. **(C, D)** Analysis of caspase 3/7 and caspase 8 activities in uninfected or ORFV NA1/11 infected (MOI=5) A549 and LLC cells at 24 hpi. The caspase activity was expressed as the relative ratios of the control cells without infection (designated as 1). Data are representative images or expressed as the mean ± SD of each group from three separate experiments. **P<0.01.

We next investigated which of cell death pathways ORFV NA1/11 infection triggered for cell death in lung cancer cells. Following infection with, or without, ORFV NA1/11, we analyzed the frequency of lung cancer cell apoptosis by flow cytometry. As shown in **Figures 3A, B**, ORFV NA1/11 infection significantly increased the percentage of apoptotic A549 and LLC cells. Furthermore, ORFV NA1/11 infection also significantly increased the levels of caspase 3/7 and caspase 8 activities in both A549 and LLC cells (**Figures 3C, D**). Co-treatment with a pan-caspase inhibitor of Z-VAD did not rescue the cells from death induced by ORFV NA1/11, but indeed delayed cell death for one day (data not shown). In addition, there was no significant increase in the relative levels of receptor interacting serine/threonine kinase 3 (RIPK3) expression in A549 and LLC cells following ORFV NA1/11 infection, compared to control cells (data not shown). Thus, ORFV NA1/11 infection induced the caspase-dependent apoptosis, but not necroptosis, in A549 and LLC cells.

### ORFV NA1/11 infection inhibits the growth of implanted lung tumors tumor-bearing mice

**Figure 4:**
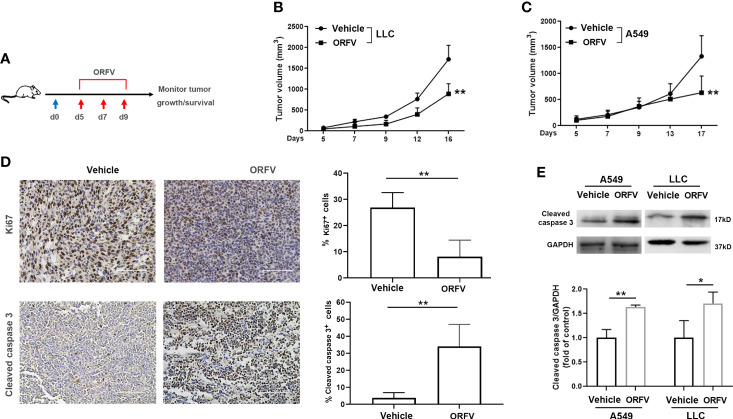
Intratumoral injection with ORFV NA1/11 inhibits tumor growth *in vivo* by promoting tumor cell apoptosis. **(A)** Treatment schema: A lung cancer model was established by subcutaneous injection with syngeneic lung cancer LLC (4× 10^5^) or A549 cells (3× 10^6^) into C57BL/6 mice or nude mice (n=6/group) at day 0 (blue arrow), respectively. When the tumors reached 50-100 mm^3^, the tumor-bearing mice were randomized and injected intratumorally with vehicle PBS as the controls or ORFV NA1/11 (red arrows). **(B, C)** The dynamic growth of LLC and A549 tumors in mice was measured longitudinally. **(D)** Representative images of tumor sections following immunohistochemical staining with anti-Ki67, or anti-cleaved caspase 3. Scale bars =100 μm. Quantitative analysis of Ki67^+^ tumor cells and cleaved caspase 3^+^ tumor cells in tumor tissues were counted in five fields (400×) from each group and the data are the average number of positive cells per field. **(E)** Western blotting analyses of the relative levels of cleaved caspase 3 in tumor tissues of mice that had been treated with PBS or ORFV NA1/11. Data are expressed as the mean ± SD of each group from three separate experiments. *P<0.05, **P<0.01.

Next, we tested the efficacy of ORFV NA1/11 oncolysis *in vivo*. After establishment of implanted lung tumors, we treated intratumorally with ORFV NA1/11 or vehicle PBS for three times (**Figure 4A**). We found that the tumor volume increased in mice with time and intratumoral injections with ORFV NA1/11 significantly reduced tumor volumes, compared with the PBS control group (**Figures 4B, C**). However, ORFV NA1/11 infection did not affect the body weights of mice (**Supplementary Figures 2A, B**) and tissue morphology (**Supplementary Figure 2C**) in mice. Moreover, Ki67 is a well-known marker of tumor proliferation in oncology and the prognosis of malignant tumors. Accordingly, we measured Ki67^+^ tumor cells by immunohistochemistry, ORFV NA1/11 treatment decreased the number of Ki67^+^ cells in the tumors, compared to PBS treatment (**Figure 4D**). HE staining analysis displayed that the number of cells in solid tumor decreased greatly, accompanied by vacuoles in many tumor cells in the ORFV NA1/11 injected tumors (data not shown). As expected, the number of cleaved caspase 3^+^ tumor cells and cleaved caspase 3 expression in tumor tissues from the ORFV NA1/11-treated mice were significantly more than that in the tumor tissues from the control mice (**Figures 4D, E**). Together, these data evidenced that the ORFV NA1/11 treatment inhibited the growth of implanted lung tumors by promoting tumor cell apoptosis.

### ORFV NA1/11 infection induces ICD

**Figure 5:**
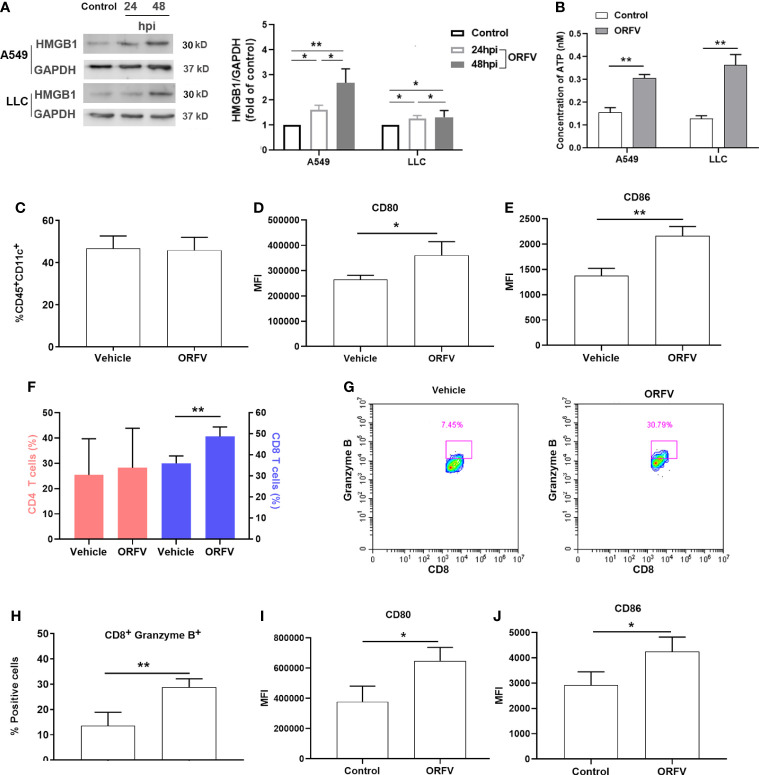
ORFV NA1/11 infection promotes the activation of DCs and recruitment of active CD8 T cells. **(A)** Western blot analysis of the cytoplasmic HMGB1 protein levels in A549 and LLC cells following infection with, or without, ORFV NA1/11 (MOI=5) for indicated time points. Quantification was performed using Quantity one software. **(B)** The extracellular levels of ATP in culture supernatants of A549 and LLC cells following infection with, or without, ORFV NA1/11 (MOI 5) were determined using an ATP Assay Kit, according to the manufacturer’s protocol. **(C)** Flow cytometry analysis of the percentages of DCs in tumors tissues. **(D, E)** Flow cytometry analyses of CD80 and CD86 expression on DCs in tumors tissues of mice, respectively. **(F)** Flow cytometry analysis of the percentages of CD4 and CD8 T cells in tumors tissues. **(G)** The percentages of CD8^+^Granzyme B^+^ T cells in tumors tissues. **(H)** Expression of granzyme B of tumor-infiltrating CD8 T cells in tumors tissues. **(I, J)** Flow cytometry analyses of CD80 and CD86 expression on the migrated DCs *in vitro*. Data are expressed as the mean ± SD of each group from three separate experiments. *P<0.05, **P<0.01.

To further explore whether ORFV NA1/11 infection induced cell death triggered ICD, we analyzed the relative protein levels of cytoplasmic HMGB1 in A549 and LLC cells following infection with ORFV NA1/11 for 24 and 48 hours by Western blot assays. HMGB1 released from the nucleus and its subsequent secretion and ATP released from dying cancer cells in the context of ICD, was described as a consensus marker for ICD (Fucikova et al., 2020). In comparison with the control cells, ORFV NA1/11 infection increased in the release of the nuclear HMGB1, evident by the increased protein levels of HMGB1 in the cytoplasm of A549 and LLC cells in a trend of time-dependent (**Figure 5A**). Moreover, ORFV infection led to a significant increase in extracellular ATP levels (**Figure 5B**). Those indicated that ORFV NA1/11 infection induced the release of ICD-associated DAMPs *in vitro*. Next, we explored how ORFV NA1/11 infection induced ICD *in vivo*. We found that although ORFV NA1/11 treatment did not significantly alter the frequency of CD11c^+^ DCs infiltrates (the gating strategy shown in **Supplementary Figure 3** and **Figure 5C**) it did significantly increase the levels of CD80 and CD86 expression in CD11c^+^ DCs in tumors (**Figures 5D, E**). These indicated that ORFV NA1/11 treatment promoted DCs activation in the tumor tissues. More importantly, ORFV NA1/11 treatment significantly increased the frequency of CD8 T cells infiltrates in the tumor tissues, relative to that of the controls (the gating strategy shown in **Supplemental Figure S3**, **Figure 5F**). There was also a significant increase in the percentage of CD8^+^Granzyme B^+^ T cells (**Figures 5G, H**) after ORFV NA1/11 treatment, indicating an activated cytotoxic phenotype. In addition, the ORFV NA1/11-infected tumor cells did not recruit significantly more DCs in transwell migration assays (co-culture strategy shown in **Supplementary Figure 4**, data not shown). However, co-culture of the ORFV NA1/11-infected tumor cells with DCs significantly enhanced CD80 and CD86 expression on DCs (**Figures 5I, J**), consistent with the notion that ORFV NA1/11-infected tumor cells enhanced the activation of surround DCs.

### ORFV NA1/11-infected tumor cells promote DC activation and CD8 T cell trafficking through the CXCL16/CXCR6 axis

**Figure 6:**
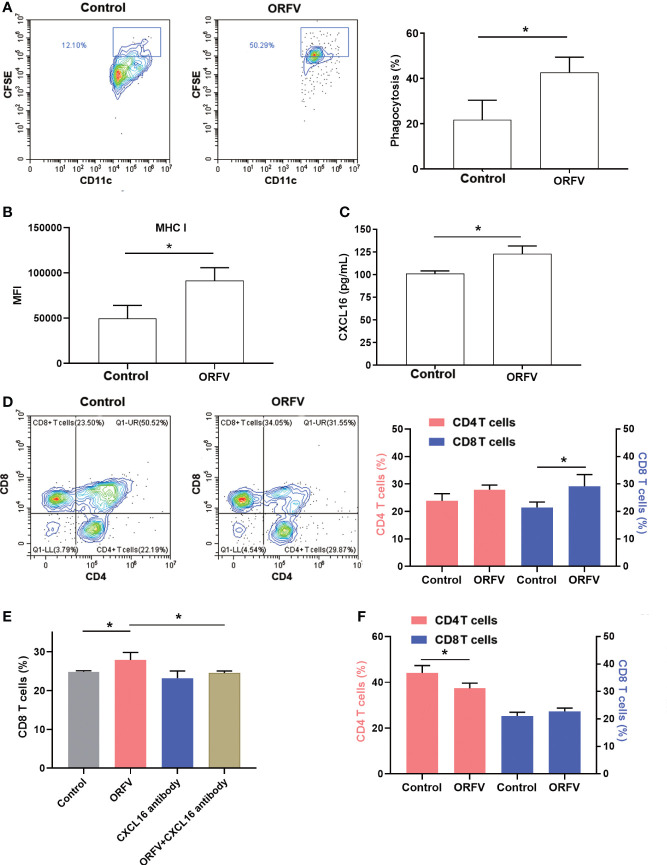
ORFV NA1/11 infection in lung cancer cells promotes DC activation and increases CD8+ T cell migration, dependent on the CXCL16/CXCR6 signaling. **(A)** Flow cytometry analysis of DC phagocytosis of CFSE-labeled tumor cells. LLC cells were labeled with CFSE and infected with, or without, ORFV NA1/11 (MOI=5). Subsequently, the CFSE-labeled tumor cells were co-cultured with DCs for 24 hours in a transwell plate. The phagocytosis of DCs (CD11c^+^CFSE^+^) was analyzed by flow cytometry. **(B)** The levels of MHC I expression in DCs were analyzed by flow cytometry after staining them with anti-MHC (I) **(C)** ELISA assays revealed that ORFV NA1/11 infection increased the levels of CXCL16 in the supernatants of cultured LLC cells. **(D)** Co-culture of DCs and ORFV NA1/11 (MOI=5) infected or uninfected LLC cells (the bottom chamber) with T cells in the upper chamber for 24 hours promoted the migration of CD8 T cells *in vitro*. **(E)** Co-culture of T cells with ORFV NA1/11 infected (MOI=5) or uninfected LLC cells for 24 hours in the presence or absence of anti-CXCL16 indicated that anti-CXCL16 treatment abrogated the ORFV-enhanced migration of CD8 T cells *in vitro*. **(F)** Co-cultured with splenic T cells from CXCR6^-/-^ mice with the ORFV NA1/11 infected or uninfected LLC cells decreased the frequency of CD4 T cell, but did not alter the frequency of CD8 T cells *in vitro*. Data are representative images or expressed as the mean ± SD of each group from three independent experiments. *P<0.05.

DCs are professional APCs and able to induce potent T cell immunity by phagocytosis, processing and presenting antigens to T lymphocytes. We first tested how DCs phagocytosed ORFV NA1/11-infected tumor cells. We found that co-culture of DCs with CFSE-labeled ORFV NA1/11-infected LLC cells significantly increased the phagocytosis of CFSE-labeled ORFV NA1/11-infected LLC cells and enhanced MHC I expression in DCs, compared with uninfected tumor cells (**Figures 6A, B**). These suggest that the enhanced DC activation by ORFV NA1/11-infected tumor cells may be more effective in presenting tumor antigen.

Next, we examined the cytokine profile in the tumor tissues by cytokine antibody arrays. We found that ORFV NA1/11 treatment significantly altered the levels of cytokines that were involved in apoptosis, autoimmunity/inflammation and angiogenesis (**Table 1**). Notably, ORFV NA1/11 treatment significantly elevated the levels of CXCL16 in the tumors. Similarly, ORFV NA1/11 infection also increased the levels of CXCL16 in the supernatants of cultured LLC cells (**Figure 6C**). Whereas co-culture of DCs with ORFV NA1/11-infected LLC cells did not alter significantly the levels of CXCL16 in the supernatants of cultured cells (data not shown), implicating that increased CXCL16 was secreted by tumor cells. Accordingly, we tested the hypothesis that ORFV NA1/11 infected tumor cells were more effective to recruit CD8 T cells, which was mediated by CXCL16. Actually, we found that co-culture of ORFV NA1/11 infected LLC cells with T cells in a transwell system significantly increased the frequency of migrated CD8 T cells from WT mice, compared with uninfected tumor cells (**Figure 6D**). The enhanced CD8 T cell migration by ORFV NA1/11 infected LLC cells was significantly mitigated by pre-treatment with anti-CXCL16 (**Figure 6E**). While co-culture of DCs or T cells with ORFV NA1/11-infected LLC cells did not significantly alter the frequency of migrated CD8 T cells from CXCR6^-/-^ mice, it did significantly decrease the frequency of migrated CD4 T cells (**Figure 6F**). Therefore, ORFV NA1/11 infection significantly elevated CXCL16 secretion by lung cancer cells, promoted DCs activation, and recruited more CD8 T cells to enhance antitumor T cell responses.

**Table 1:** The antibody array data of differentially expressed cytokines.

Cytokine	Mean (ORFV,n=3)	Mean (NC,n=3)	p-value	Fold chang	Regulation	Functional Classification	
TREM-1	11.98	11.38	0.1821	1.52	up	Chemokine
TPO	8.01	8.43	0.0146	0.75	down	Growth factor
KC	14.80	12.46	0.0380	5.05	up	Angiogenesis+ Autoimmunity/Inflammation
CXCL16	10.59	8.73	0.0036	3.64	up	Chemokine
Pentraxin 3	12.12	11.22	0.0039	1.87	up	Apoptosis + Autoimmunity/Inflammation
CCL21	0.00	4.32	0.0325	0.05	down	Chemokine
G-CSF	12.50	9.43	0.0423	8.42	up	Growth factor
CRP	11.52	10.29	0.0425	2.34	up	Immunity/Inflammation
RAGE	10.70	9.93	0.0472	1.71	up	MAPK signaling + p53/TP53 signaling + NF-kB
C5a	15.87	13.01	0.0008	7.25	up	Inflammation
L-Selectin	13.51	12.74	0.0012	1.71	up	Tumor Metastasis
IL-2 Ra	8.94	7.97	0.0033	1.95	up	Tyrosine kinase signaling pathway
IL-17F	7.20	2.27	0.0282	30.41	up	Inflammation
MIP-2	12.87	9.96	0.0285	7.53	up	Chemokine
MIP-3a	7.87	6.70	0.0318	2.25	up	Chemokine
Resistin	13.08	11.74	0.0036	2.53	up	Insulin1 signaling

Triggering receptor expressed on myeloid cells-1, TREM-1.

Thrombopoietin, TPO.

Chemokine (C-C motif) ligand 21, CCL21.

Keratinocyte-derived chemokine, KC.

Granulocyte Colony Stimulating Factor, G-CSF.

C-reaction protein, CRP.

Complement component 5a, C5a.

Macrophage inflammatory protein, MIP.

Recombinant receptor for advanced glycation endproducts, RAGE.

## Discussion

ORFV is a promising and relatively safe therapeutic reagent for malignant tumors, because ORFV infection usually induces self-limited lesions in the skin (Spyrou and Valiakos, 2015). Hence, ORFV may be a relatively safe oncolytic virus. Moreover, ORFV infection can induce potent cellular immune responses by promoting the expression of inflammatory cytokines (Friebe et al., 2004 **;** Friebe et al., 2011), including IFN, IL-2, and TNF, but does not mount strong neutralizing antibody responses (Hosamani et al., 2009). Actually, infection with a living ORFV induces immune responses in multiple murine models of cancers such as melanoma, colorectal cancer and breast cancer, and also has an oncolytic effect on human lung cancer in a lung cancer xenograft model (Rintoul et al., 2012).

Our previous studies have isolated a strain of ORFV NA1/11 from tissue of a sheep collected from a farm located in Jilin Province in northeastern China (Li et al., 2012), and have identified its entire genome by sequencing (Li et al., 2015). In the current studies, we explored therapeutic potential of ORFV NA1/11 for lung cancer. We found that ORFV NA1/11 infection caused lung cancer cell death varying from 20% to about 50%. Subsequently, we found that ORFV NA1/11 infection resulted in A549 and LLC cell apoptosis. These findings indicate that these cells express receptors, such as SERP1/PABPC4-like proteins for virus binding to enter (Chen et al., 2021). The dose-dependent cytotoxicity against malignant lung tumor cells demonstrated that ORFV NA1/11 had OV activity.

To understand how ORFV NA1/11 infection caused lung cancer cell apoptosis, we tested whether ORFV NA1/11 infection modulated cell cycle status in lung cancer cells. We found that ORFV NA1/11 infection induced cell cycle arrest in G2/M phase and reduced the relative levels of cyclin B1 expression in both A549 and LLC cells. These suggest that ORFV NA1/11 infection may alter the cell cycling of lung cancer cells to benefit its replication, similar to many DNA viruses, such as papillomavirus (Reinson et al., 2015), simian virus (Rohaly et al., 2010) and adenovirus (Jiang et al., 2015) as well as others (Chen et al., 2004 **;** Yuan et al., 2005 **;** Dove et al., 2006). More importantly, cell cycle arrest is associated with apoptosis. Hence, ORFV NA1/11 infection induced cell cycle arrest, leading to apoptosis of lung cancer cells.

After virus infects a cell, the virus can interact with some cellular proteins to avoid cell rapid death, thus promoting virus replication and release, although the virus infection can eventually kill the host cells (Ma et al., 2020). Our data indicated that ORFV NA1/11 infection triggered the apoptosis of lung cancer cells by increasing caspase 3/7/8 activity, suggesting that ORFV NA1/11 infection-induced lung cancer cell apoptosis was caspase-dependent. Similarly, ORFV NA1/11 treatment also efficiently inhibited the growth of implanted lung cancer and increased the levels of cleavage caspase 3 expression in the tumor tissues, indicating that ORFV NA1/11 treatment promoted lung cancer cell apoptosis in the tumors. Thus, the oncolytic effect of ORFV NA1/11 on lung cancer cell death is mediated by inducing their apoptosis.

Under normal circumstances, immune cells usually do not recognize apoptotic cells well, which is a unique strategy for protecting host against pathogens (Minute et al., 2020). In tumor immunotherapy field, it is important to understand whether the mode of cell death is tolerogenic or immunogenic (Ma et al., 2020). APCs can migrate towards and phagocytose pyroptotic and autophagic tumor cells. We found that apoptotic tumor cells induced by ORFV NA1/11 infection increased the relative protein levels of cytoplasmic HMGB1 in A549 and LLC cells. This result suggests that ORFV-NA1/11 infection may release DAMP molecules, which can promote ICD. The released HMGB1 can bind to receptors (TLR2, TLR4, TLR9, TIM3 and RAGE) on APCs to activate them, including DCs maturation (Russell and Barber, 2018). APCs are also able to capture antigens of dead cells and their debris and present tumor antigens to T cells to initiating and regulating T cell immunity. Indeed, co-culture of DCs with ORFV NA1/11-infected tumor cells engulfed more tumor cells than with uninfected tumor cells, and DCs exhibited high levels of CD80, CD86 and MHC I expression. Furthermore, ORFV therapy enhanced the accumulation of effector CD8^+^Granzyme B^+^ T cells in the tumors.

CXCL16, a chemokine, can recruit CXCR6^+^ cells. The levels of CXCL16 and CXCR6 are detected in various cancers and correlated with both better and worse survival, dependent on tumor types (La Porta, 2012). CXCL16^-/-^ mice display deficient CD8 T cell responses (Wein et al., 2019). CXCL16 can function in promoting interactions between DCs and CD8 T cells as well as effector T cell trafficking (Matloubian et al., 2000). Radiation-induced CXCL16 release by breast cancer cells attracts effector T cells (Matsumura et al., 2008). We found that ORFV NA1/11 infection enhanced CXCL16 expression and secretion in lung cancer cells and co-culture of T cells from WT mice, but not from CXCR6^-/-^ mice, with ORFV NA1/11-infected lung cancer cells promoted the migration of CD8 T cells in a transwell experimental system. Furthermore, ORFV NA1/11 treatment recruited more CD8 T cells in the lung tumors in a CXCL16-dependent manner. However, we found that co-culture of T cells from either WT or CXCR6^-/-^ mice with ORFV NA1/11-infected lung cancer cells greatly affected CD4 T cell migration regardless of their sources. Additionally, co-culture of T cells with ORFV NA1/11-infected lung cancer cells decreased the number of migrated CD4^+^CD8^+^ T cells. Possibly, other chemokines may be important for the migration of different subsets of T cells.

Induction of ICD by an OV is mediated by action of OV as an *in situ* cancer vaccine because the dying tumor cells induced by an OV can release tumor antigens and inflammatory molecules as adjuvants that are recognized by APCs to present tumor antigens to specific T cells, leading to antitumor T cell immunity (Russell and Barber, 2018 **;** van Vloten et al., 2018). ORFV infection induces tumor cell apoptosis that facilitates phagocytosis of dying tumor cells by DCs and recruits CD8 T cells through the CXCL16/CXCR6 axis. Therefore, our results may provide new insights into the mechanisms by which ORFV infection of tumor cells induces antitumor T cell immunity. Our findings may aid in design of new ORFV-based therapies for lung cancer patients.

## Data availability statement

The raw data supporting the conclusions of this article will be made available by the authors, without undue reservation.

## Ethics statement

The animal study was reviewed and approved by Institutional Animal Care and Use Committee of Foshan University.

## Author contributions

RW, SL, and FL contributed to study design and data interpretation. RW, JM, XL, GZ performed the *in vivo* and virological experiments; RW, JM, and XL performed the molecular biological experiments. RW wrote the manuscript, SL, and FL edited the manuscript. All authors have read and approved the final version of the manuscript.

## Funding

This study was supported by grants from the National Natural Science Foundation of China (No. 81773271), the Education Department of Guangdong Province (2018KQNCX284), the Joint Fund of Basic and Applied Basic Research Fund of Guangdong Province (2019A1515110689). The funding sources had no role in the study design, data collection, data analysis, interpretation or writing of the report.

## Conflict of interest

The authors declare that the research was conducted in the absence of any commercial or financial relationships that could be construed as a potential conflict of interest.

## Publisher’s note

All claims expressed in this article are solely those of the authors and do not necessarily represent those of their affiliated organizations, or those of the publisher, the editors and the reviewers. Any product that may be evaluated in this article, or claim that may be made by its manufacturer, is not guaranteed or endorsed by the publisher.

## Supplementary material

Supplementary Figure 1 ORFV NA1/11 infection causes varying levels of lung cancer cell death *in vitro*. **(A)** The indicated lung cancer cells were infected with, or without, ORFV NA1/11 at an MOI of 5 for 48 hours. Their viability was measured by WST-8 assays. Data are expressed as the relative percentages of cell viability in each group of cells from three separate experiments. The cell viability in the control cells without virus infection was designated as 100%. **(B)** The A549 cell were infected with ORFV NA1/11-GFP at an MOI of 5. The images were obtained 48 hpi.

Supplementary Figure 2 Intratumoral injection with ORFV NA1/11 does not change the body weights and many tissue **(A, B)** Longitudinal measurements of body weights in both groups of tumor-bearing mice. **(C)** Histological analysis of the morphology of the indicated tissues after H&E staining. Data are representative images or expressed as the mean ± SD of each group (n=6) of mice.

Supplementary Figure 3 Flow cytometry gating strategies. All cells in each group were gated on single mononuclear cells and on living cells. Subsequently, the living CD11c^+^ DCs, CD4 T cells, CD8 T cells were gated and the levels of CD80, CD86 and granzyme B expression were analyzed.

Supplementary Figure 4 A diagram of experimental design: LLC cells were infected with ORFV NA1/11 for 2 h and washed. DCs were overlaid onto infected tumor cells and cultured in transwell chambers.
