# Supplementary figures and images for: ORFV infection enhances CXCL16 secretion and causes oncolysis of lung cancer cells through immunogenic apoptosis
Source: Front Cell Infect Microbiol. 2022 Jul 25;12:910466. doi: 10.3389/fcimb.2022.910466 (PMC9358046; doi:10.3389/fcimb.2022.910466)

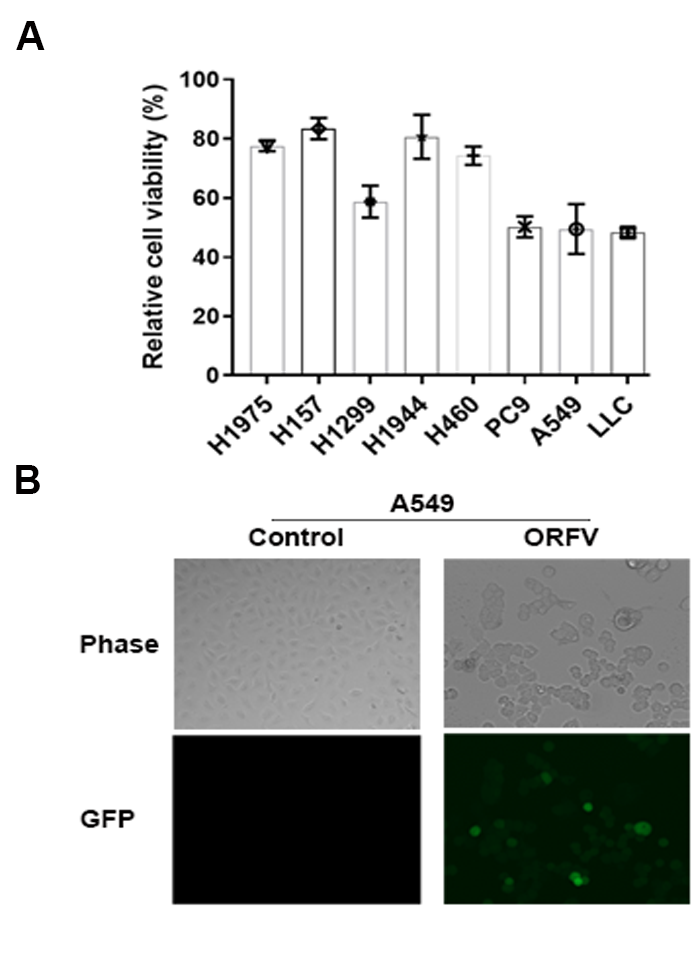

Supplement: Supplementary Figure 1 — ORFV NA1/11 infection causes varying levels of lung cancer cell death in vitro. (A) The indicated lung cancer cells were infected with, or without, ORFV NA1/11 at an MOI of 5 for 48 hours. Their viability was measured by WST-8 assays. Data are expressed as the relative percentages of cell viability in each group of cells from three separate experiments. The cell viability in the control cells without virus infection was designated as 100%. (B) The A549 cell were infected with ORFV NA1/11-GFP at an MOI of 5. The images were obtained 48 hpi. [file Image_1.tif]

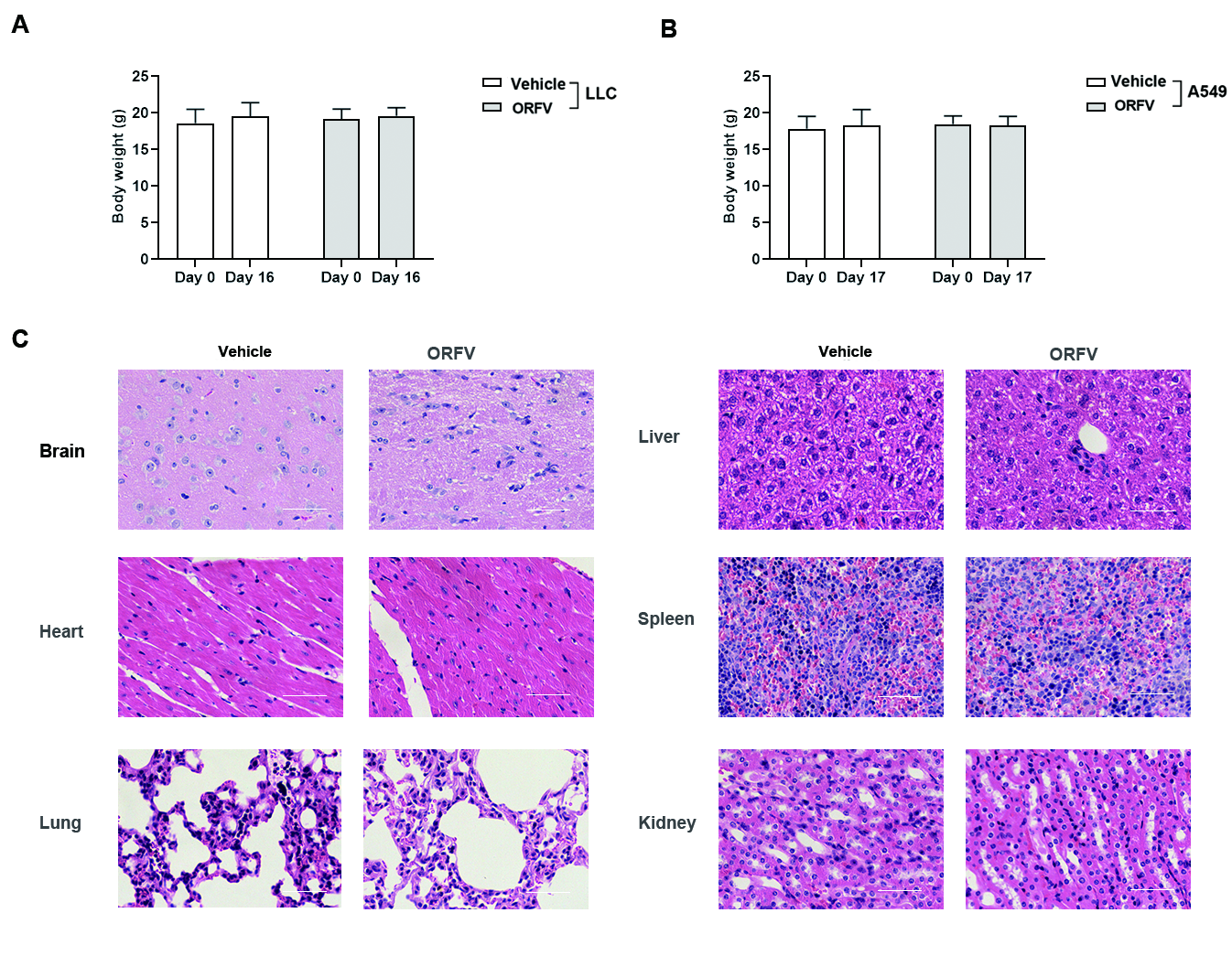

Supplement: Supplementary Figure 2 — Intratumoral injection with ORFV NA1/11 does not change the body weights and many tissue (A, B) Longitudinal measurements of body weights in both groups of tumor-bearing mice. (C) Histological analysis of the morphology of the indicated tissues after H&E staining. Data are representative images or expressed as the mean ± SD of each group (n=6) of mice. [file Image_2.tif]

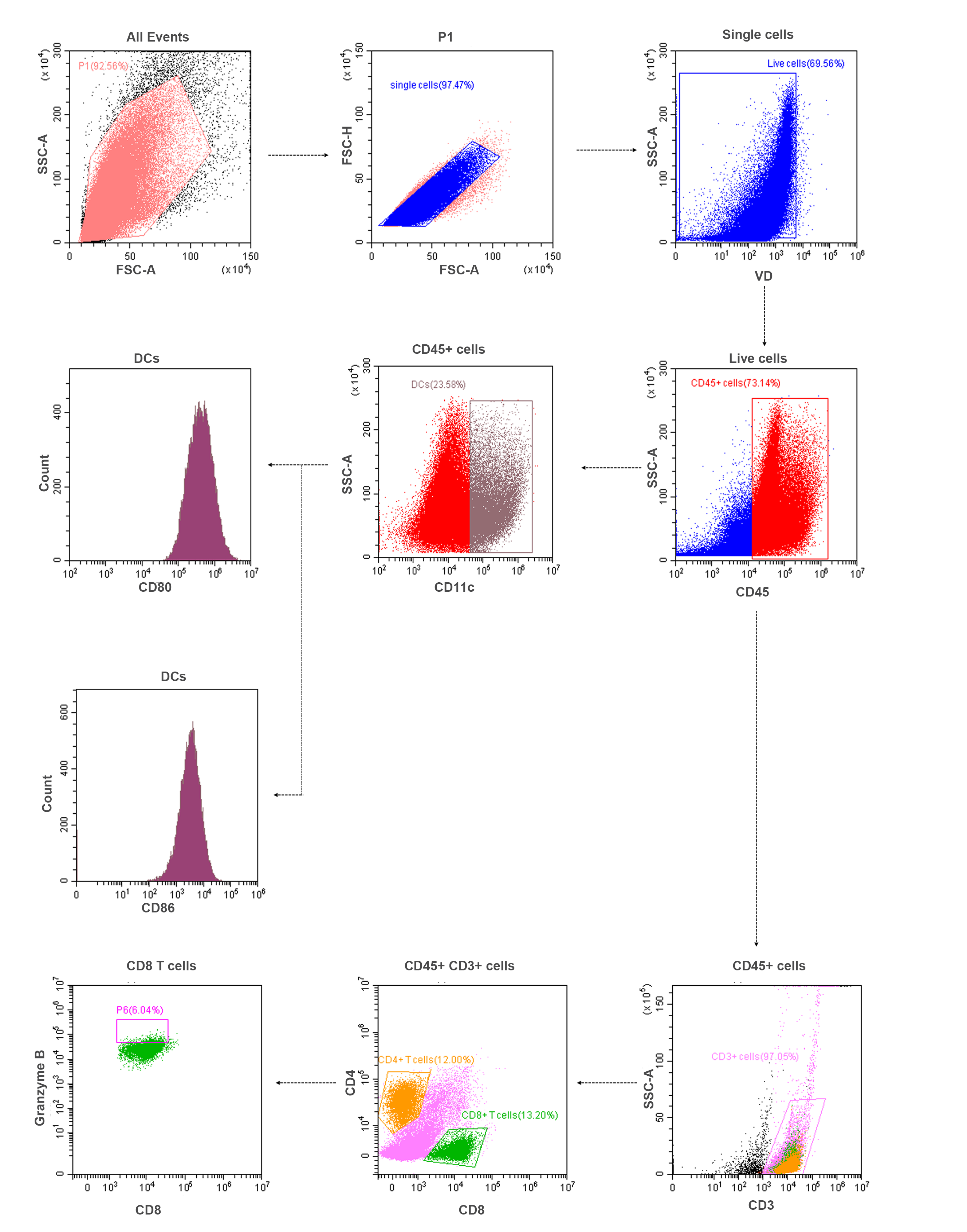

Supplement: Supplementary Figure 3 — Flow cytometry gating strategies. All cells in each group were gated on single mononuclear cells and on living cells. Subsequently, the living CD11c+ DCs, CD4 T cells, CD8 T cells were gated and the levels of CD80, CD86 and granzyme B expression were analyzed. [file Image_3.tif]

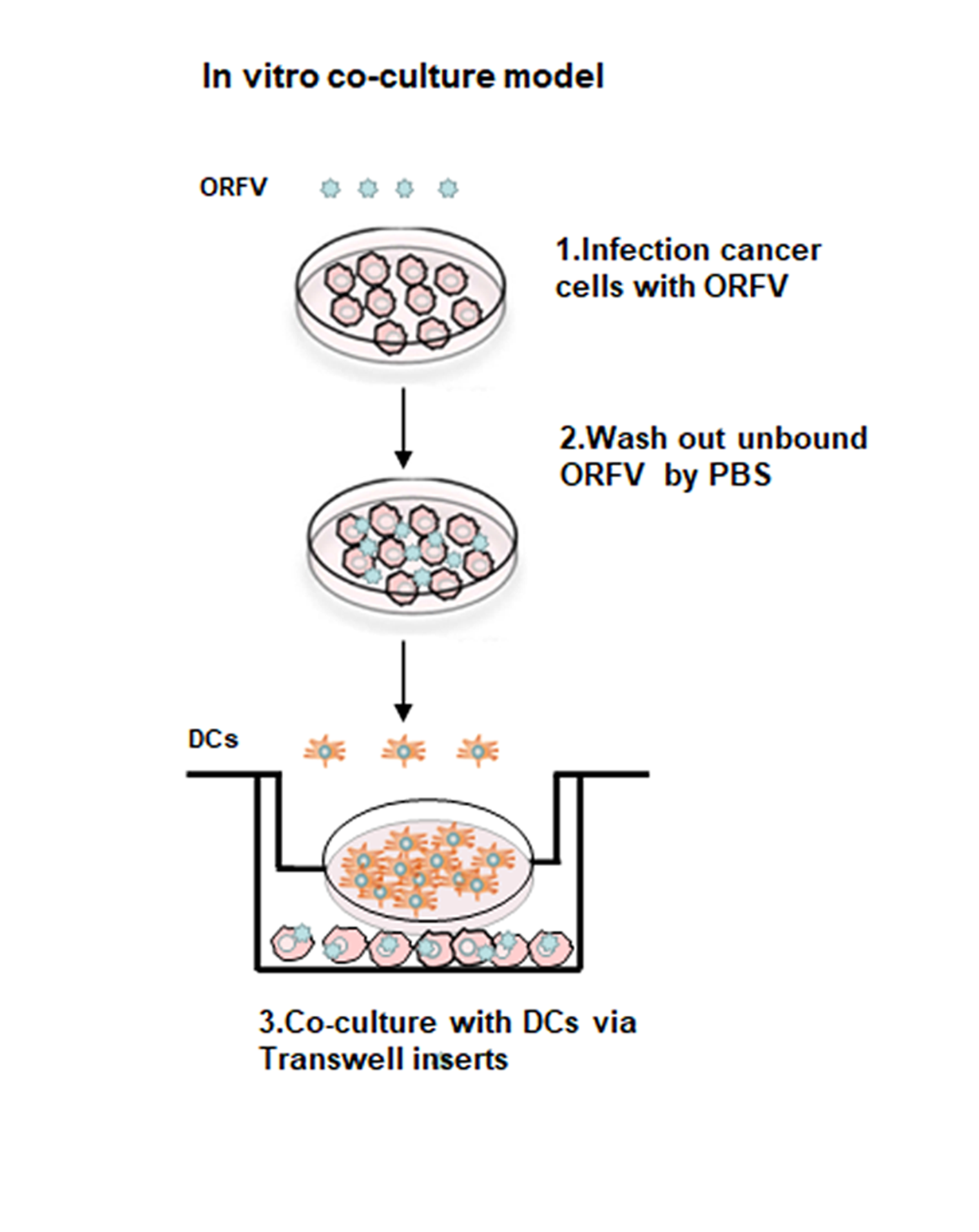

Supplement: Supplementary Figure 4 — A diagram of experimental design: LLC cells were infected with ORFV NA1/11 for 2 h and washed. DCs were overlaid onto infected tumor cells and cultured in transwell chambers. [file Image_4.tif]
